# Early Systemic Granulocyte-Colony Stimulating Factor Treatment Attenuates Neuropathic Pain after Peripheral Nerve Injury

**DOI:** 10.1371/journal.pone.0043680

**Published:** 2012-08-24

**Authors:** Po-Kuan Chao, Kwok-Tung Lu, Yun-Lin Lee, Jin-Chung Chen, Hung-Li Wang, Yi-Ling Yang, Mei-Yun Cheng, Ming-Feng Liao, Long-Sun Ro

**Affiliations:** 1 Department of Life Science, National Taiwan Normal University, Taipei, Taiwan; 2 Division of Neuromuscular Disorders, Department of Neurology, Chang Gung Memorial Hospital and University, Chang-Gung University, Tao-Yuan, Taiwan; 3 Department of Pharmacology, Chang-Gung University, Tao-Yuan, Taiwan; 4 Department of Physiology, Chang-Gung University, Tao-Yuan, Taiwan; 5 Institute of Biotechnology, National Chia-Yi University, Chia-Yi, Taiwan; Universidad Federal de Santa Catarina, Brazil

## Abstract

Recent studies have shown that opioid treatment can reduce pro-inflammatory cytokine production and counteract various neuropathic pain syndromes. Granulocyte colony-stimulating factor (G-CSF) can promote immune cell differentiation by increasing leukocytes (mainly opioid-containing polymorphonuclear (PMN) cells), suggesting a potential beneficial role in treating chronic pain. This study shows the effectiveness of exogenous G-CSF treatment (200 µg/kg) for alleviating thermal hyperalgesia and mechanical allodynia in rats with chronic constriction injury (CCI), during post-operative days 1–25, compared to that of vehicle treatment. G-CSF also increases the recruitment of opioid-containing PMN cells into the injured nerve. After CCI, single administration of G-CSF on days 0, 1, and 2, but not on day 3, relieved thermal hyperalgesia, which indicated that its effect on neuropathic pain had a therapeutic window of 0–48 h after nerve injury. CCI led to an increase in the levels of interleukin-6 (IL-6) mRNA and tumor necrosis factor-α (TNF-α) protein in the dorsal root ganglia (DRG). These high levels of IL-6 mRNA and TNF-α were suppressed by a single administration of G-CSF 48–144 h and 72–144 h after CCI, respectively. Furthermore, G-CSF administered 72–144 h after CCI suppressed the CCI-induced upregulation of microglial activation in the ipsilateral spinal dorsal horn, which is essential for sensing neuropathic pain. Moreover, the opioid receptor antagonist naloxone methiodide (NLXM) reversed G-CSF-induced antinociception 3 days after CCI, suggesting that G-CSF alleviates hyperalgesia via opioid/opioid receptor interactions. These results suggest that an early single systemic injection of G-CSF alleviates neuropathic pain via activation of PMN cell-derived endogenous opioid secretion to activate opioid receptors in the injured nerve, downregulate IL-6 and TNF-α inflammatory cytokines, and attenuate microglial activation in the spinal dorsal horn. This indicates that G-CSF treatment can suppress early inflammation and prevent the subsequent development of neuropathic pain.

## Introduction

During nerve injury, pain is associated with release of pro-inflammatory cytokines such as tumor necrosis factor (TNF)-α, interleukin (IL)-1β, and IL-6, which are essential for establishing nociceptive processing [1], [2]. Thus, the attenuation of pro-inflammatory cytokines alleviates neuropathic pain. Opioids are quite effective in fighting acute and chronic pain. In addition, opioids help regulate the immune system [3] and have neuroprotective properties [4]. Nevertheless, clinical exogenous opioid administration is associated with several side effects in addition to tolerance development because of their central mechanisms of action, thus limiting their use [5]. To overcome these limitations, endogenous opioid-mediated antinociception has been extensively studied, and its physiological and clinical relevance have been established [6].

In both early inflammation and chronic neuropathic models, hyperalgesia can be partially counteracted by a local antinociceptive system involving opioid-containing leukocytes [6]–[10]. Under inflammatory conditions, leukocytes secrete opioid peptides that bind to opioid receptors on peripheral sensory neurons and mediate antinociception [11]–[15]. In humans, opioid peptides locally released by leukocytes can decrease pain intensity as well as the consumption of pain medication under post-surgical stress conditions [6].

Majority of the opioid-containing leukocytes during early inflammation are polymorphonuclear (PMN) cells [12], [16]. Treatment with granulocyte-colony stimulating factor (G-CSF) causes hematopoietic stem cell egression from bone marrow niches and mobilization to the peripheral blood [17]. The G-CSF receptor (G-CSFR) is a transmembrane protein expressed on cells of the neutrophil lineage, including progenitor and differentiating myeloid cells in the bone marrow and mature neutrophils in the peripheral blood [18]. G-CSF then initiates precursor cell proliferation and differentiation into mature PMN cells [19]. Endogenous CRF [20], [21] and chemokines (ex. CXCL2/3) [22] expressed in inflamed tissue are prominent agents that trigger opioid peptide release from leukocytes, thereby inhibiting pain. Thus, G-CSF is an important factor for inducing the generation of new PMN cells, suggesting a potential beneficial role for treating inflammatory and chronic pain. We hypothesized that PMN cells and their recruitment by G-CSF may be effective in alleviating neuropathic pain induced by nerve injury. Therefore, we proposed to administer G-CSF to an animal model with neuropathic pain and evaluate whether G-CSF-induced activation of PMN cells can reverse chronic pain by releasing peripheral endogenous opioids.

**Figure 1:**
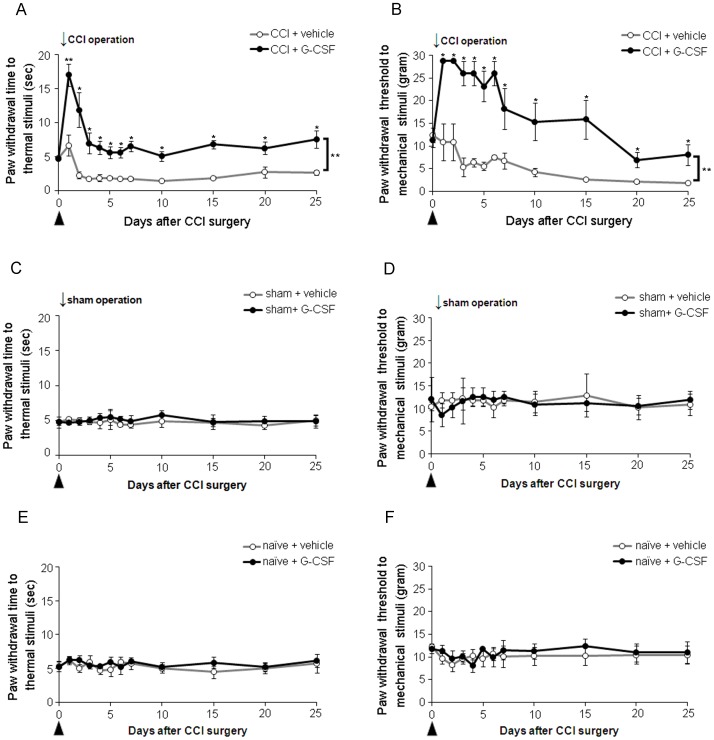
G-CSF alleviates long-term thermal hyperalgesia and mechanical allodynia in rats with CCI. (A) Thermal hyperalgesia was significantly developed in CCI rats treated with vehicle, compared to the pre-operation (*p*<0.01) and sham-operation (*p*<0.05) status and persisted throughout the experimental period (25 days). In contrast, G-CSF treatment significantly attenuated the development of thermal hyperalgesia from 1 to 25 days post-CCI compared to the vehicle treatment control. (B) Mechanical allodynia was significantly developed compared to the pre-operation status and persisted throughout the experimental period in CCI rats treated with vehicle; G-CSF treatment alleviated mechanical allodynia from 1 to 25 days post-CCI compared to the vehicle treatment control. (C, D) Compared to vehicle treatment, G-CSF treatment did not alter thermal and mechanical responses in sham-operated rats. (E, F) Similarly, no effect was observed throughout the experimental period on thermal and mechanical responses in naïve control rats treated with G-CSF compared to those treated with vehicle. Data are shown as the means±SEM, n = 6 per group, two-way repeated measures ANOVA, *p<0.05, **p<0.01. Arrows indicate the CCI operation times; arrowheads indicate the time of injection of G-CSF or vehicle.

## Methods

### Subjects

Adult male Sprague–Dawley rats (BioLASCO Taiwan Co., Ltd. Taipei, Taiwan) weighing around 200–250 g were used. The animal room was artificially lit from 6:00 h to 18:00 h. Three rats were housed in each cage in a temperature-controlled (24°C) animal colony; pellets of rat chow and water were available *ad libitum*. All behavioral procedures took place during the light cycle. All procedures were conducted in accordance with the Guidelines for Care and Use of Laboratory Animals and were approved by the Institutional Animal Care and Use Committee (IACUC) at the National Taiwan Normal University. Every effort was made to minimize animal suffering as well as the number of animals required to generate reliable experimental data.

**Figure 2:**
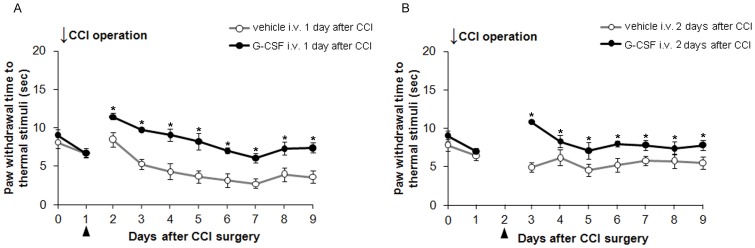
G-CSF demonstrates a therapeutic time window (0–48 h) after nerve injury. One-day (A) and two-day (B) delayed injection of G-CSF significantly reversed thermal hyperalgesia compared to the vehicle control, which maintained less thermal hyperalgesia during the entire experiment period. Data are shown as the means±SEM, n = 6 per group, two-way repeated measures ANOVA, *p<0.05. Arrows indicate the CCI operation times; arrowheads indicate the G-CSF or vehicle injection time.

### Surgical Procedures

All experimental procedures were performed on rats that were deeply anesthetized with sodium pentobarbital (50 mg/kg body weight, intraperitoneal (i.p.) injection). Sterile operating instruments were used. Briefly, 4 ligatures of 4–0 chromic gut were loosely tied around the nerve with approximately 1.0–1.5 mm interval between the knots such that epineural circulation was preserved. At the time of tying, the ligatures just barely reduced the nerve diameter [23]. Sham operations were also carried out under the effect of anesthesia to expose, but not manipulate, the right sciatic nerve. The left sides of all rats were not subjected to any surgery.

**Table 1:** Effects of G-CSF on mobilized bone marrow cells in the peripheral blood.

Hours afterCCI surgery	0	3		6		12		24		48		72		144	
Groups	Naïve	CCI+vehicle	CCI+G-CSF	CCI+vehicle	CCI+G-CSF	CCI+vehicle	CCI+G-CSF	CCI+vehicle	CCI+G-CSF	CCI+vehicle	CCI+G-CSF	CCI+vehicle	CCI+G-CSF	CCI+vehicle	CCI+G-CSF
**Total WBCs**	4.72 (0.23)	6.04 (0.73)	10.73 (1.06)*	6.45 (0.81)	11.11 (1.02)*	8.72 (2.03)	15.76 (2.22)*	6.84 (0.82)	13.95 (1.19)*	6.31 (1.53)	11.29 (0.67)*	5.76 (0.98)	10.88 (3.31)	4.88 (0.75)	5.80 (1.07)
**PMNs**	4.16 (0.18)	5.18 (0.61)	9.88 (1.10)*	5.18 (0.70)	10.43 (0.99)*	7.81 (2.12)	14.53 (2.36)*	5.85 (0.87)	12.51 (1.21)*	4.71 (1.28)	9.40 (0.64)*	4.41 (1.11)	9.55 (3.24)	3.82 (0.60)	4.48 (0.61)
**Lymphocytes**	0.41 (0.10)	0.66 (0.13)	0.69 (0.12)	1.04 (0.28)*	0.38 (0.08)	0.58 (0.24)	0.62 (0.35)	0.58 (0.33)	0.80 (0.26)	1.38 (0.53)	1.40 (0.33)	1.09 (0.44)	1.13 (0.88)	1.15 (0.12)	0.99 (0.35)
**Monocytes**	0.04 (0.02)	0.09 (0.09)	0.08 (0.07)	0.10 (0.06)	0.06 (0.04)	0.08 (0.03)	0.40 (0.28)	0.30 (0.12)	0.41 (0.09)	0.16 (0.21)	0.34 (0.14)	0.15 (0.17)	0.11 (0.22)	0.39 (0.09)	0.21 (0.14)

Compared to vehicle treatment in rats with CCI, G-CSF treatment in rats with CCI significant increased the number of circulating WBCs and PMN cells at 3, 6, 12, 24, and 48 h after CCI. In contrast, the levels of circulating lymphocytes and monocytes at the above-mentioned time points were not significantly different between the G-CSF- and vehicle-treated rats with CCI, except at 6 h when there was a transient increase in the level of lymphocytes in vehicle group. Data are shown as the mean±SEM; n = 6 per group; Mann–Whitney *U* test, *p<0.05.

**Figure 3:**
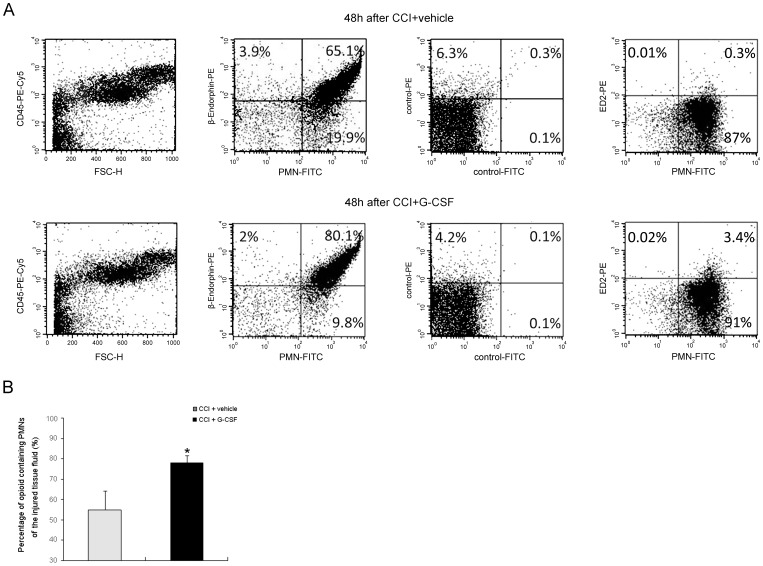
Effects of G-CSF on mobilized immune cells in the tissue fluid around the injured nerves. Immune cell subpopulations of tissue fluid were quantified by flow cytometry. (A) At 48 h, increased expression of markers, such as, PMN cell marker (stained using anti-PMN-FITC), and β-endorphin marker (stained using anti-β-endorphin-PE), on leukocytes is observed in G-CSF–treated rats with CCI than in vehicle-treated rats with CCI (n = 6 per group). CD45-PE-Cy5 and ED2-PE antibodies were used to react with all hematopoietic cells and macrophages, respectively. Low staining with the respective control antibodies, confirmed a specific antibody staining of opioid-containing PMN cells. In contrast, low staining with ED2-PE and PMN-FITC antibodies (0.3–3.4%), confirmed a specific staining of PMN cells by PMN-FITC antibody. (B) At 48 h, the number of β-endorphin–containing PMN cells in G-CSF–treated rats with CCI is significantly higher than that in vehicle-treated rats with CCI. Data are shown as the mean±SEM, n = 6 per group; *t* test, *p<0.05.

### Protocols of Drug Treatments

Postoperative intravenous (i.v.) G-CSF injections (200 µg/kg Figrastim; Kyowa Hakko Kirin, Japan) were given to examine the effect on neuropathic pain. Six rats received chronic constriction injury (CCI) plus an immediate single i.v. G-CSF injection and 6 received CCI plus an immediate single i.v. vehicle (normal saline) injection. Twenty-four rats were used to examine whether G-CSF alters pain thresholds in sham and/or naïve animals; 6 (12 total) each received a single injection of G-CSF (naïve or sham + G-CSF, respectively) and the same number received a single injection of vehicle (naïve or sham + vehicle, respectively). Thirty-six rats were used to examine the effect of G-CSF on neuropathic pain responses and determine its therapeutic window; each of the 6 rats received CCI plus i.v. G-CSF or vehicle 24, 48, and 72 h after operation.

**Figure 4:**
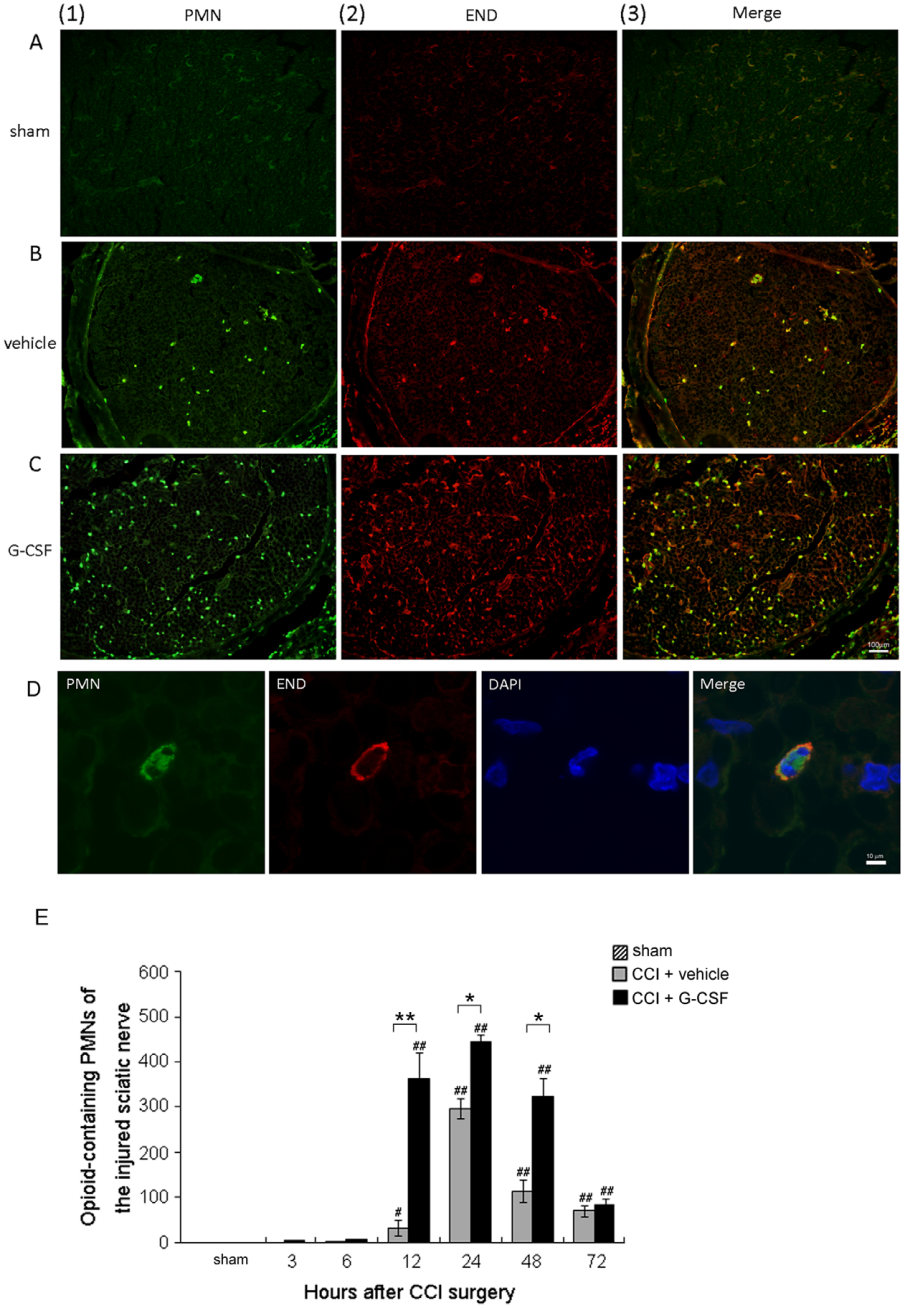
G-CSF treatment increases the number of migrated β-endorphin-containing PMN cells in injured nerves. (A–C) Confocal laser scanning microscopy of PMN cells and β-endorphin peptides in the right sciatic nerve of vehicle-treated sham rats (A1–3), vehicle-treated CCI rats (B1–3), and G-CSF treated CCI rats (C1–3) 12 h after CCI, respectively. No apparent PMN cells or β-endorphin peptides were observed in the vehicle-treated sham operation rats. However, at 12 h, the count of β-endorphin-containing PMN cells was significantly higher in the G-CSF-treated CCI rats than in the vehicle-treated CCI rats. Scale bar: 100 µm. (D) Confocal high-power field laser scanning pictures showing a PMN cell with a typical segmented nucleus, which stained positive for β-endorphin peptides. Green: PMN cells; red: β-endorphin (END)-containing cells; blue (DAPI): cell nuclei. Scale bar: 10 µm. (E) The number of β-endorphin-containing PMN cells per section as calculated at different time points. The count of β-endorphin-containing PMN cells was higher in the CCI rats treated with G-CSF (black bars) compared to those treated with vehicle (grey bars) between 12–48 h after nerve injury. Data are shown as the means±SEM, n = 5 per group; one-way ANOVA, *p<0.05, **p<0.01: CCI + G-CSF group compared to CCI + vehicle group; #*p*<0.05, ##*p*<0.01: CCI + G-CSF or CCI + vehicle group compared to vehicle-treated sham operation control.

To examine the counteractive effects of naloxone methiodide (NLXM; Sigma, St. Louis, MO) in G-CSF-induced antinociception experiments, G-CSF was injected alone or with NLXM in each of the 6 rats. Three days after CCI, rats under ether anesthesia received NLXM (30 µg) in a total volume of 180 µL at the same site of nerve injury. A polyethylene tube was placed 1 cm from the tip around a 26 G needle to ensure the same depth of needle insertion into the middle of the scar tissue after CCI. The injection site was reproducibly covered by approximately 1 cm of the nerve, including the ligation site and sites proximal and distal to it [9].

**Figure 5:**
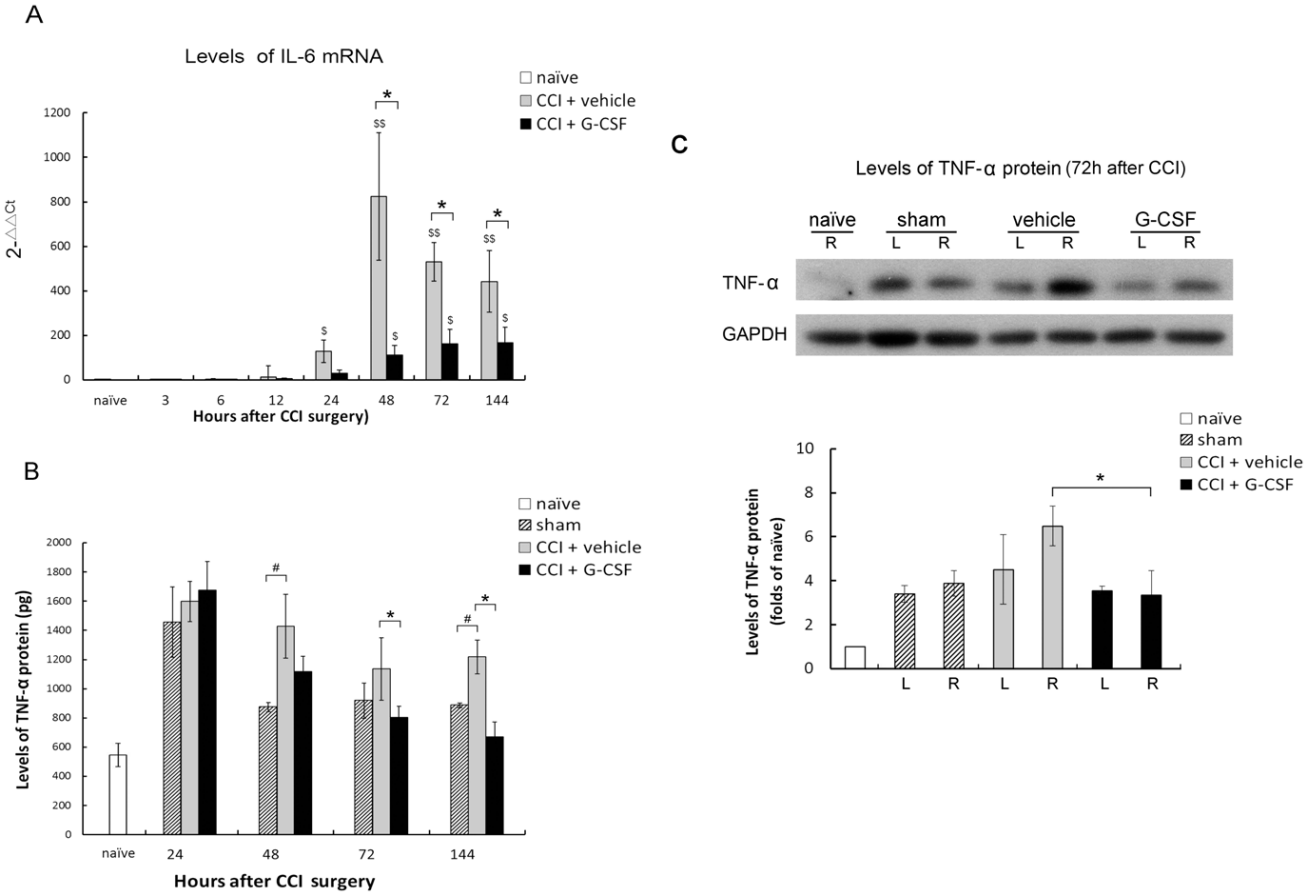
G-CSF reduces the levels of IL-6 mRNA and TNF-α protein in the DRG after CCI. (A) Quantitative real-time PCR revealed significantly lower levels of IL-6 mRNA in G-CSF-treated CCI rats than in those treated with vehicle 48 and 144 h after nerve injury. Data are shown as the means±SEM, n = 5 per group; one-way ANOVA, *p<0.05: CCI + G-CSF group compared to CCI + vehicle group; $*p*<0.05, $$*p*<0.01: CCI + G-CSF or CCI + vehicle group compared to naïve controls. (B) ELISA analysis showed significantly lower levels of TNF-α in G-CSF–treated rats with CCI than in vehicle-treated rats with CCI at 72 and 144 h after nerve injury. Data are shown as the mean±SEM, n = 5 per group; one-way ANOVA, *p<0.05: CCI + G-CSF group compared to CCI + vehicle group; #*p*<0.05: CCI + G-CSF or CCI + vehicle group compared to vehicle-treated sham-operated controls. (C) Western blot analysis showed significantly lower levels of TNF-α in G-CSF–treated rats with CCI than in vehicle-treated rats with CCI at 72 h after nerve injury. Data are shown as the mean±SEM; n = 5 per group; *t* test, *p<0.05: CCI + G-CSF group compared to CCI + vehicle group. R: right side; L: left side.

### Behavioral Tests for Thermal Hyperalgesia and Mechanical Allodynia

#### Habituation

Prior to testing, each animal was placed for a 10 min habituation period in a test box with the dimensions of 30×30×15 cm^3^ having 3 mirrored sides to minimize stress. No food or water was available to the rats during the experiment. Each animal was used only once and was euthanized at the end of the experiment by administering a lethal dose of pentobarbital [24].

**Figure 6:**
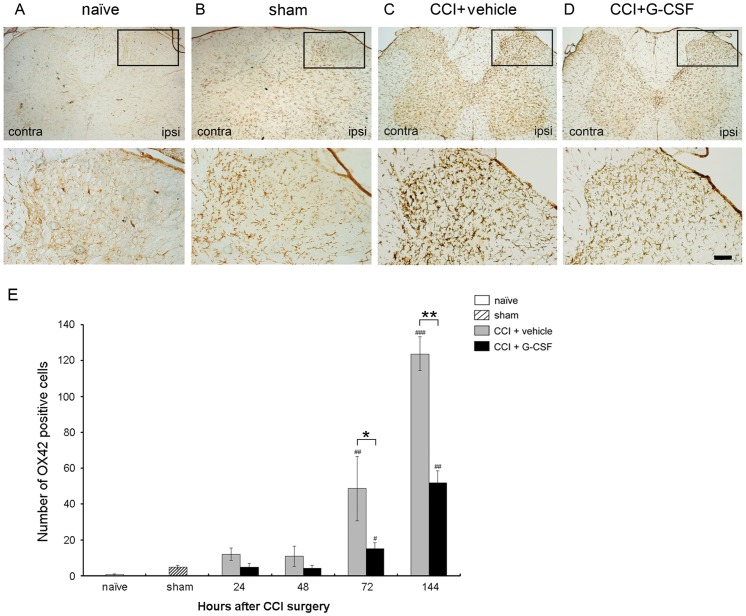
G-CSF reduces immunoreactivity of OX-42 in the spinal cord 72 h after CCI. Representative images show the OX-42 peptides in the right L5 dorsal horn of (A) naïve, (B) sham operation rats, (C) vehicle-treated CCI rats, and (D) G-CSF-treated CCI rats. There was a basal and mild activation of OX-42 immunoreactivity in naïve and sham operation rats, respectively. A significantly higher level of OX-42 immunoreactivity was observed in CCI rats treated with vehicle. In contrast, a significantly lower level of OX-42 immunoreactivity (similar to the sham-operation rats) was noted 72 h post-CCI in the CCI rats treated with G-CSF than those treated with vehicle. (E) Quantification of OX-42 immunoreactivity in L5 dorsal horn after vehicle- or G-CSF- treated CCI rats 72 h post-CCI. Data are shown as the means±SEM, n = 5 per group; one-way ANOVA, *p<0.05, **p<0.01: CCI + G-CSF group compared to CCI + vehicle group; #*p*<0.05, ##*p*<0.01: CCI + G-CSF or CCI + vehicle group compared with naïve or sham operation controls. ipsi: ipsilateral (right) side; contra: contralateral (left) side. Scale bar: 50 µm.

#### Thermal hyperalgesia test

To avoid bias, surgery and behavioral testing were performed by 2 different investigators. CCI-induced thermal hyperalgesia was measured by the latency of hind paw withdrawal from a hot water bath (YIHDER Water Bath BH-230) (46°C) stimulus. The rat was gently held in a towel and the hind paw immersed in hot water. Paw-withdrawal values were obtained before and after the surgical operation and intravenous injections of G-CSF or normal saline. A cut-off time of 20 s was imposed to avoid tissue injury. The average of 3 readings (allowing 10 min intervals between paw withdrawals to prevent sensitization) for paw withdrawal was calculated.

**Figure 7:**
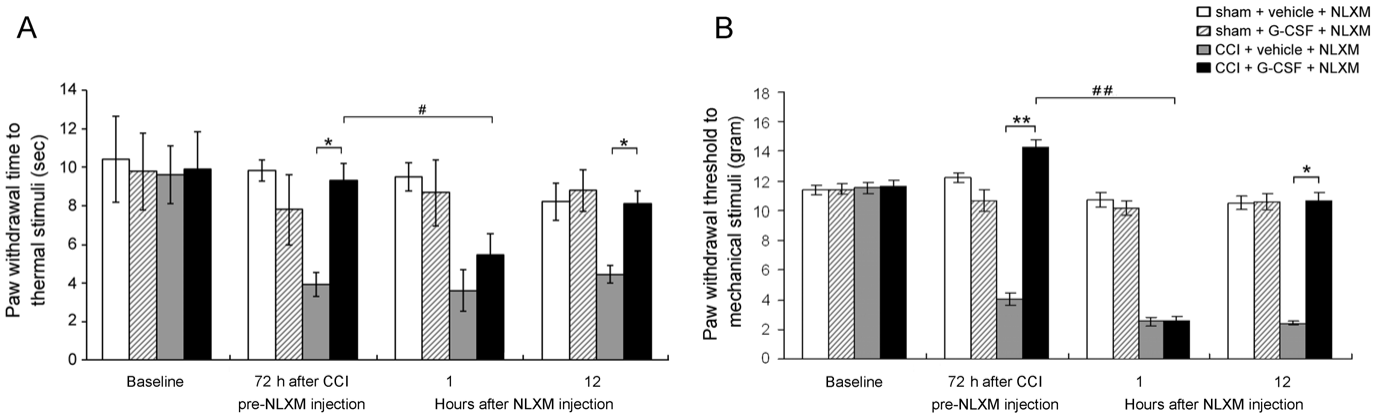
NLXM reverses the G-CSF anti-nociceptive effect. The histograms represent the effect of opioid/opioid receptor interaction on thermal and mechanical responses in sham or CCI rats with/without G-CSF treatment 72 h post-CCI operation, followed by a local injection of NLXM. One hour after injection, NLXM reversed G-CSF antinociception effect on both thermal hyperalgesia (A) and mechanical allodynia (B); however, the effect of NLXM lasted for 1 h but did not show any further reversal effects on antinociception at 12 h post-injection. In contrast, NLXM did not alter the thermal and mechanical responses in sham-operated rats with/without G-CSF treatment. Data are shown as the means±SEM, n = 6 per group; two-way repeated measures ANOVA, *p<0.05, **p<0.01: CCI + G-CSF group compared to CCI + vehicle group; #*p*<0.05, ##*p*<0.01: CCI + G-CSF group compared to CCI + G-CSF + NLXM.

#### Mechanical allodynia test

Mechanical allodynia was assessed by von Frey hair, according to a previously described protocol [25]. von Frey hairs were applied to the central region of the plantar surface of a hind paw in ascending order of force (0.7, 1.2, 1.5, 2.0, 3.6, 5.5, 8.5, 11.7, 15.1, and 29 g). Each filament was applied 5 times. When the rats showed 1 withdrawal response to a given filament, the bending force of that filament was defined as the mechanical threshold intensity. The median threshold intensity was calculated from the values following 1 descending and 2 ascending trials. The experimental conditions were identical for both naïve and experimental rats. Behavioral testing resumed a day after the operations and continued for 25 consecutive days.

**Figure 8:**
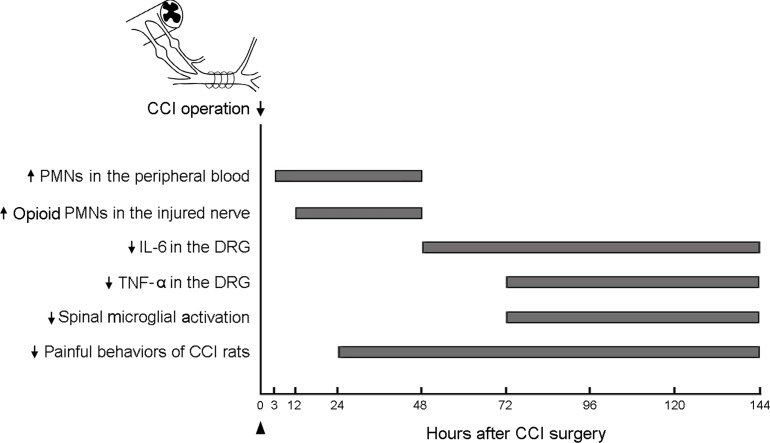
The time sequence of the effects of G-CSF on pain, cytokine levels, and microglial activation. The sequential effect of G-CSF in rats with CCI are po (post-operative) increase in PMN cells and opioid-containing PMN cells in the peripheral blood at 3 h and in the injured nerves at 12 h, downregulation of the levels of IL-6 within po 48–144 h and TNF-α within po 72–144 h in the DRG, and suppression of spinal microglial activation within po 72–144 h, all of which are associated with long-term pain alleviation after nerve injury. The arrow indicates the time at which the operation for CCI was performed; the arrowhead indicates the G-CSF injection time.

### Blood Cell Counting and Flow Cytometry

For cell counting, we used K_3_ EDTA VACUTAINER blood collection tubes (BD Biosciences, Heidelberg, Germany) to collect heart blood from deeply anesthetized rats (n = 6 rats per group) and analyzed with a semi-automated hematology analyzer (SYSMEX F820).

Cell suspensions were prepared from the surgical sites and stained as follows: tissue fluid (2 days after CCI, 100 µL) was collected from deeply anesthetized rats, fixed and lysed as described by the FACS lysing solution® kit (BD Biosciences), and pressed through a 70 mm nylon filter. For intracellular stains, the cells were fixed with 1% paraformaldehyde and permeabilized with saponin buffer (0.5% saponin, 0.5% bovine serum albumin, and 0.05% NaN_3_ in phosphate-buffered saline (PBS); Sigma). Permeabilized cells were incubated with different primary antibodies, and the samples were stained using mouse anti-rat CD45 -phycoerythrin (PE) cyanine dye 5, and incubated with a conjugated polyclonal antibody (CD45; 1∶500; BD PharMingen, CA) for labeling all leucocytes. To differentiate the leukocyte subpopulations, cell suspensions were stained with fluorescein isothiocyanate (FITC)-conjugated rabbit anti-rat polyclonal antibody recognizing granulocytes (PMN; 1∶500; Accurate Chemical, USA) and PE-conjugated mouse anti-rat macrophages monoclonal antibody (ED2; 1∶500; AbD Serotec, United Kingdom). For intracellular staining, the cells were prepared and incubated with rabbit anti-rat β-endorphin-PE polyclonal antibody that can recognize opioid peptides (β-endorphin; 1∶500; Bioss). The specificity of the staining was verified by incubating cell suspensions with the appropriate isotype-matched control antibodies. To calculate the absolute number of PMN cells around the stained cell suspension, 10,000 fluorescence-activated cell sorting (FACS) events were acquired. All obtained data were analyzed using CellQuest software (BD Biosciences, USA).

### RNA Extraction and Reverse Transcription PCR (RT-PCR)

Lumbar 5 (L5) dorsal root ganglia (DRG) were excised from naïve rats and rats with or without G-CSF treatment (n = 5 per group) 3, 6, 12, 24, 48, 72 and 144 h after CCI. L5 DRG samples were frozen in liquid nitrogen, and then ground to a fine powder by using a mortar and pestle. The total RNA of L5 DRG was extracted with TRIzol® reagent, according to the manufacturer’s instructions. RNA was converted into cDNA using a first strand cDNA synthesis kit (cat. 18080-051; Invitrogen) with random hexamers.

### Quantitative RT-PCR

Quantitative RT-PCR was carried out in a final volume of 25 µL comprising optimal concentrations of primers and probes in 96-well plates on an ABI PRISM 7000 detector (Applied Biosystems, Foster City, CA). The primers and probes were purchased from Applied Biosystems and delivered as 20× concentrates. The reaction mixture for RT-PCR comprised 12.5 µL of Taqman® Universal PCR Master Mix containing 1.25 U of AmpliTaq DNA polymerase, 1.25 µL of probes, 1–2 µg of L5 DRG cDNA, and water to a final volume of 25 µL. TaqMan® rat IL-6 MGB probes (cat. Rn99999011_m1) were labeled with a FAM reporter fluorescent dye and TaqMan® rat GAPDH MGB probes (P/N 4352338E), with a VIC reporter fluorescent dye. The amplification started with 2 min at 50°C, 10 min at 95°C, followed by 50 cycles of the following: 15 s at 95°C and 1 min at 60°C. Amplicons were run in di-duplicate in a separate tube assay for the quantification of IL-6 gene in comparison to the assays for the internal control gene glyceraldehyde 3-phosphate dehydrogenase (GAPDH). ΔCt represents the mean Ct value of each sample and was calculated for both IL-6 and GAPDH. The relative fold change of IL-6 expression in naïve rats in L5 DRG was calculated by the comparative Ct method (2^−(ΔΔCt)^) of relative quantification.

### Enzyme-linked Immunosorbent Assay (ELISA) and Western Blotting Assay

We used enzyme-linked immunosorbent assay (ELISA) and western blot analysis to examine the effect of G-CSF on the TNF-α level. Forty-eight rats were used for this analysis, of which 16 were subjected to CCI and received an immediate single i.v. G-CSF injection, 16 were subjected to CCI and received vehicle injection, and 16 underwent surgery without CCI and received vehicle injection. Four rats from each group were killed at 24, 48, 72, and 144 h after operation. L5 and L6 DRG were resected and briefly sonicated in ice-cold buffer (50 mM Tris-HCl [pH 7.8], 50 mM NaCl, 10 mM ethylene glycol tetraacetic acid [EGTA], 5 mM EDTA, 2 mM sodium pyrophosphate, 4 mM para-nitrophenylphosphate, 1 mM sodium orthovanadate, 1 mM phenylmethylsulfonyl fluoride (PMSP), 20 ng/mL leupeptin, and 4 ng/mL aprotinin). The samples were ultracentrifuged at 13,600 rpm for 30 min at 4°C. Bradford assay was performed with the lysates to ensure that an equal quantity of protein was loaded into each well.

The L5 samples of the experimental group were subsequently analyzed in duplicates in a double-blinded manner. TNF-α levels in the soluble fraction of DRG were measured by ELISA using a commercially available kit, according to the manufacturer instructions (Biosource/Invitrogen, Carlsbad, CA, USA).

In the western blotting assay, equal protein loading was verified using Coomassie staining after sodium dodecyl sulfate polyacrylamide gel electrophoresis (SDS-PAGE). Protein samples were separated by SDS-PAGE and transferred onto polyvinylidene fluoride (PVDF) membranes. The blots were blocked using 5% bovine serum albumin (BSA) in Tris-buffered saline (TBS) with 0.5% Tween-20 overnight at 4°C and then incubated with TNF-α (1∶2000; Millipore, Billerica, MA, USA) or GAPDH (internal control; 1∶2500; Alpha Diagnostic International, San Antonio, TX, USA) primary antibody in TBS containing 1% BSA and 0.5% Tween-20 overnight at 4°C. This step was followed by incubation with horseradish peroxide (HRP)-linked secondary antibody (1∶2500, anti-rabbit IgG; Cell Signaling Technology) for 45 min at room temperature. All washing was performed using TBS containing 0.1% Tween-20. The bands were detected using an enhanced chemiluminescence western blotting analysis system (RPN 2108; Amersham International, Amersham, UK).

### Immunohistochemistry

Rats were deeply anesthetized with sodium pentobarbital and transcardially perfused with PBS (Sigma), followed by a fixative solution containing 4% paraformaldehyde. Spinal cord segments (L4–L6) and sciatic nerves were resected and placed in 4% paraformaldehyde for 4 h and transferred to 30% sucrose at 4°C overnight. The samples were subsequently embedded in OCT compound (Tissue-Tek 4583; Sakura, Tokyo, Japan) and rapidly frozen. Tissue sections of 10 µm thickness were obtained using a freezing microtome (CM 3050; Leica, Nussloch, Germany), mounted on polylysine-coated slides. After rinsing with cold PBS (0.1 M, pH 7.4), the sections were heated in citrate buffer for 20 min at 90°C for antigen retrieval. In microglial response states, after incubation of protein-blocking buffer (BioGenex, San Ramon, CA) for 1 h at room temperature, the sections were incubated at 4°C overnight with a mouse anti-rat CD11B primary antibody (OX-42 clone, 1∶1000 dilution; Millipore). After rinsing, the sections were assayed with the Super Sensitive Polymer-HRP IHC Detection System (BioGenex), according to the manufacturer’s instructions. Peroxidase activity was developed by treatment with 3,3′-diaminobenzidine for 2 min. In immunofluorescence analysis, the sections were blocked with protein-blocking buffer (BioGenex, San Ramon, CA) and incubated with rabbit anti-β-endorphin antibody (1∶200; Chemicon, Neuromics, Minneapolis, MN) for 24 h at 4°C, followed by the secondary antibody (1∶200, TRITC-conjugated goat anti-rabbit IgG; Jackson Immuno Research Laboratories, Inc., USA) for 1 h at room temperature. After β-endorphin immunostaining, the sections were further processed with PMN immunostaining with a rabbit anti-rat PMN-FITC antibody (1∶200; Accurate Chemical, NY) [26] for 2 h at room temperature, as described previously [27]. The Images were acquired using a confocal spectral microscope (Leica TCS-SP2, Heidelberg, Germany) connected to digital camera and computer; montages were created and analyzed using Image-ProPlus 5.0 (Media Cybernetics Inc., Silver Spring, MD). The areas occupied by the stained tissues were highlighted and measured for each section of sciatic nerve (n = 5 per group, 5 sections per animal) and expressed in terms of the density (number/µm^2^) of the right sciatic nerve. Omission of the primary antibody served as the negative control for all experiments.

### Statistical Analysis

All statistical comparisons were made using SPSS version 15.0 (SPSS Inc., Chicago, IL). Normally distributed data were analyzed by Student’s *t*-test (for 2 groups) or one-way analysis of variance (ANOVA) (for 3 or more groups). If the data lacked equal variance or normality, Mann–Whitney rank sum test or Kruskal–Wallis test was used. Data derived from behavioral experiments were analyzed by repeated measures two-way ANOVA across testing time points to detect overall differences across different treatment groups. In both cases, when significant main effects were detected, *post hoc* Newman–Keuls test was performed to determine the source of the difference. Data are expressed as mean±standard error of the mean (SEM). A *p* value less than 0.05 was considered significant.

## Results

After unilateral CCI of the sciatic nerve, animals developed thermal hyperalgesia (Fig. 1A; from day 2 to day 25) and mechanical allodynia (Fig. 1B; from day 3 to day 25) of the right injured hindpaw compared to naïve (two-way repeated-measures ANOVA, *p*<0.01) or sham-operated rats (two-way repeated-measures ANOVA, *p*<0.05). Post-CCI thermal hyperalgesia and mechanical allodynia were not altered by the infusion of the vehicle (saline); in contrast, CCI rats with i.v. injection of G-CSF exhibited significantly attenuated thermal hyperalgesia and mechanical allodynia compared to the rats treated with the vehicle (*p*<0.01) (Fig. 1A, B). However, no significant alterations in thermal or mechanical sensations were observed when G-CSF was injected into sham-operated animals (Fig. 1C, D). A lack of effects on thermal and mechanical sensations was observed in naïve animals who received either G-CSF or vehicle (Fig. 1E, F).

To determine the therapeutic window of G-CSF and to determine whether G-CSF is effective in reversing thermal hyperalgesia once established, delayed G-CSF injections were administered 24, 48, and 72 h after CCI. Delayed G-CSF injections (24 and 48 h after CCI) resulted in a significant reversal of thermal hyperalgesia compared to the effect of only vehicle treatment (*p*<0.05) (Fig. 2A, B). These findings are similar to those in rats with immediate G-CSF injections, which significantly attenuated thermal hyperalgesia. However, delayed G-CSF injections 72 h after CCI did not significantly attenuate thermal hyperalgesia (data not shown). Thus, the effect of G-CSF on the attenuation of thermal hyperalgesia of CCI rats has a therapeutic window of 0–48 h after nerve injury.

Cell counting analysis of immune cell subpopulations in peripheral blood showed that the count of specific immune cells significantly increased in CCI rats treated with G-CSF compared to those that received vehicle treatment (Table 1). At 3 to 48 h after nerve injury, CCI rats treated with G-CSF exhibited increased total counts of WBCs (1.72- to 2.03-fold) and PMN cells (1.86- to 2.14-fold) in the peripheral blood compared to those treated with vehicle only. The count of circulating lymphocytes and monocytes increased but not significantly (Table 1). Multi-color flow cytometry, was used to examine opioid peptide expression and expression of PMN cell markers. On comparing the extent of migration of immune cells (CD45 marker cells) into the tissue fluid (Fig. 3A) around the injury sites in G-CSF- and vehicle-treated CCI rats, we found that the number of opioid containing PMN cells in the G-CSF-treated CCI rats was greater than that in the vehicle-treated CCI rats (Fig. 3A). To clarify the specificity of the PMN cell marker, we used ED2-PE to stain immune cells of the tissue fluid around the injured nerves. We found only a negligible amount of cells showing ED2-PE and PMN-FITC co-staining, indicating anti-PMN-FITC antibody is specific for PMN labeling. Furthermore, the percentages of opioid-containing leukocytes were significantly different in the tissue fluids between the 2 groups (Fig. 3B; number of opioid-positive cells in the total PMN cells, vehicle control: 55.8%±9.4% and G-CSF: 79.2%±3.5%; statistical analysis by *t* test, *p*<0.05). The animals without inflammation (naïve) were not analyzed because of lack of tissue fluid in the nerve tissue.

Nerve sections from the sciatic nerves were obtained at 3, 6, 12, 24, 48, and 72 h after CCI and were double stained with polyclonal anti-β-endorphin and anti-PMN cell antibodies. The immunohistochemical photomicrographs revealed that the majority of PMN cells also expressed β-endorphin, which was more prominent from 12 to 48 h after injury in CCI rats treated with G-CSF than in those treated with the vehicle (Fig. 4A*–*C). Increased migration of PMN cells was observed only in the injury sites (Fig. 4B–D) but not in the sham-operated nerves (Fig. 4A). After CCI, the number of opioid-containing PMN cells at 12–48 h in G-CSF-treated rats was significantly higher than that in vehicle-treated rats; further, the number of PMN cells increased earlier in G-CSF-treated rats than in vehicle-treated rats (Fig. 4E).

Pro-inflammatory cytokines, such as IL-6 and TNF-α, have been implicated in the development of hyperalgesia or allodynia after nerve injury. Real-time PCR revealed that the IL-6 mRNA expression in the L5 DRG of the injured sides of CCI rats increased 24 h after injury, and the increase persisted until 144 h after CCI. Compared to vehicle treatment, G-CSF treatment downregulated IL-6 expression within 48–144 h after CCI (Fig. 5A). ELISA showed that TNF-α (Fig. 5B) levels of G-CSF-treated rats increased 24 h after operation, whereas protein expression in the DRG was attenuated at 72 and 144 h after the operation. The peak levels in the vehicle-treated groups were higher than those in the G-CSF-treated groups. There were no differences in the peak levels between the sham-operated group and G-CSF-treated groups. Furthermore, western blotting assay performed at 72 h after the operation showed that the TNF-α protein expression in the DRG was attenuated by G-CSF treatment (Fig. 5C). However, throughout experimental period, TNF-α protein expression did not vary in the internal control samples.

In separate groups of animals, sections from the spinal cord obtained 72 h after CCI were stained with OX-42 to detect activated microglia. The expression patterns of OX-42 immunoreactivity in the dorsal horn of naïve, sham, vehicle- and G-CSF-treated rats were compared (Fig. 6A*–*D). In CCI rats treated with vehicle, the microglial cells were markedly activated in the ipsilateral dorsal horn 72 h after surgery (Fig. 6C). In contrast, CCI rats treated with G-CSF (similar to sham rats) exhibited significantly lower levels of OX-42 immunoreactivity compared to the vehicle-treated rats 72 h after nerve injury (Fig. 6D). Increased activated microglia numbers was noted in the spinal cord dorsal horn. Microgliosis was defined as cells displaying hypertrophy of cell bodies, and retraction of processes, with overlap individual microglia. There is evidence that the activated microglia numbers increased after CCI with vehicle treated but attenuated after G-CSF injection (Fig. 6E).

After unilateral CCI of the sciatic nerve, animals developed thermal hyperalgesia and mechanical allodynia. In contrast, CCI rats treated with G-CSF exhibited normal thermal and mechanical responses compared to the CCI rats treated with vehicle 72 h after operation. Interestingly, G-CSF-treated CCI rats rapidly redeveloped thermal hyperalgesia (Fig. 7A; *p*<0.05) and mechanical allodynia (Fig. 7B; *p*<0.01) after NLXM injection at that time; however, NLXM-regulated analgesia-blocked effect lasted for a maximum of 1 h, after which the G-CSF-treated CCI rats returned to their original normal thermal and mechanical response state. Thus, the analgesic effects of G-CSF on thermal hyperalgesia and mechanical allodynia are opioid/opioid receptor interaction dependent.

## Discussion

The major finding of this study is that exogenously applied G-CSF is effective in alleviating thermal hyperalgesia and mechanical allodynia in rats with CCI mainly through the activation of leukocyte-derived endogenous opioid secretion, downregulation of IL-6 and TNF-α inflammatory cytokines, and decreased microglial cell activation in the spinal dorsal horn. Prolonged inflammation increases the number of opioid-containing immune cells, tissue endorphin contents, and the efficacy of pain control [9], [28]–[31]. In particular, we found that G-CSF can alleviate neuropathic pain, at least through the following mechanisms: (1) G-CSF increases circulating and peripheral PMN cells, which are the main opioid-containing leukocytes, in early inflammation; (2) under early inflammatory conditions, the recruited PMN cells secrete opioid peptides that bind to opioid receptors on peripheral sensory neurons to mediate antinociception [8], [13], [16], [20], [32]; (3) G-CSF inhibits inflammatory cytokines that contribute to inflammatory pain; and (4) G-CSF significantly decreases microglial cell activation in the spinal dorsal horn compared to vehicle treatment. This is the first study demonstrating that G-CSF is effective in alleviating thermal hyperalgesia and mechanical allodynia in an animal model of chronic pain. Consequently, our research may introduce a new method of treating chronic neuropathic pain.

This study indicates that G-CSF is a safe drug for antinociception. Firstly, endogenous G-CSF is usually generated at the site of inflammation and acts as an endocrine hormone to mobilize immune cells from the bone marrow to replace the inflammatory cells consumed in an inflammatory reaction [33]. In G-CSF receptor knockout mice, the G-CSF-stimulated mobilization of neutrophils and hematopoietic progenitors from the bone marrow to the blood was markedly impaired [34]. Thus, G-CSF is a unique and specific receptor-dependent cytokine that drives proliferation of certain specialized hematopoietic cells, and survival and neutrophilic differentiation of myeloid progenitor cells [19], [35], [36]. Secondly, PMN cells become activated during early inflammation and are recruited by the upregulation of corticotropin-releasing factor (CRF) and chemokines of the CXC family [12], which are secreted by inflamed tissues and immune cells and may be the source of peripheral (or endogenic) endorphin [37]. Cell counting revealed that about 12 h after operation, G-CSF increases the number of PMN cells by a larger amount and in a faster manner in blood circulation and injured nerves compared to vehicle treatment. The result indicates that G-CSF may exhibit the powerful function of recruiting PMN cells while not affecting other hematopoietic cells during the early stage of inflammation (Table 1). Furthermore, there is no evidence that PMN cells secrete large amounts of β-endorphin in normal circulation, suggesting that PMN cells only secrete β-endorphin under certain circumstances. Moreover, G-CSF does not affect nociceptive responses in naïve and sham animals, which was also observed in our study (Fig. 1C–F). Our findings indicate that G-CSF acts only on the injured sciatic nerve, and is an effective targeted therapy for treating neuropathic pain.

We also show here that G-CSF increases the migration of opioid-containing PMN cells to sites of nerve injury and attenuates thermal hyperalgesia and mechanical allodynia. Although the precise underlying mechanisms remain unclear, G-CSF treatment induces peripheral analgesia probably via the secretion of opioid peptides from PMN cells. Secretion of opioids can also be stimulated by cold water swim stress or local injection of CRF and can subsequently activate the peripheral sensory neurons [38]. Therefore, G-CSF treatment may lead to an increase in the number of opioid-containing PMN cells, which can secrete opioids and attenuate neuropathic pain. Moreover, the functional integrity of the immune system is essential for peripheral antinociception. In addition, endorphins help regulate the immune system and have neuroprotective properties [15], [39].

Cytokines appear to induce acute-phase protein synthesis and are therefore a sensitive marker of tissue injury. Development of neuropathic pain is associated with multiple changes in gene expression in the DRG, which are modulated by inflammatory cytokines [40]. In particular, IL-1, IL-6, and TNF-α play important roles in neuropathic pain in humans [3] by promoting the progression of secondary injury in the acute phase of nerve injury [41], [42]. Moreover, IL-6 and TNF-α can activate microglial cells in the dorsal horn [1]. G-CSF can suppress the *in vitro* expression of inflammatory cytokines and modulate their expression in experimental allergic encephalitis [43]. In our study, IL-6 mRNA and TNF-α protein expression were suppressed in the G-CSF-treated group compared to the vehicle-treated group, which is consistent with the above findings. The DRG IL-6 mRNA and TNF-α protein levels increased immediately after the operation and then decreased slowly. Our results suggest that the relatively low inflammatory cytokine protein expression is related to less inflammation and pain.

Sensitization of the spinal dorsal horn neurons leads to prolonged enhancement of pain behaviors that can be evoked by intense C-fiber stimulation, tissue inflammation, and peripheral nerve injury [44]. There is evidence that OX-42 immunoreactivity is increased in the rat spinal cord following peripheral nerve injury in a time course that correlates with pain behaviors. Since microglia produce cytokines that are known to play roles in the central sensitization of the spinal cord, it is reasonable to suspect that microglial activation plays an important role in nociceptive processing. In this study, G-CSF inhibited central microglial activation, which significantly contributes to neuropathic pain (Fig. 6). A significantly lower level of OX-42 immunoreactivity was observed in CCI rats treated with G-CSF than in those treated with vehicle 72 h after nerve injury, indicating that G-CSF also has an anti-microglial activation effect on the spinal dorsal horn.

G-CSF may act by increasing the secretion of endogenous opioids, which in turn act on the opioid receptors of injured nerves. Our study showed similar findings, because local application of NLXM could block the G-CSF-related analgesic effects. Our results demonstrate that the effect of G-CSF on alleviating thermal hyperalgesia and mechanical allodynia is opioid/opioid receptor interaction-dependent; however, NLXM blocks the antinociception effects of G-CSF, which last for only 1 h, indicating that the alleviation of thermal hyperalgesia and mechanical allodynia by G-CSF is mediated through a continuous and complex mechanism.

In contrast, some studies have shown that G-CSF could not produce significant antinociception in inflammatory pain, cancer pain, or neuropathic pain in animal models [45], [46]. However, many aspects of the differences between those studies and ours require discussion. (1) The injection methods (subcutaneous [s.c.] versus i.v.) and injection time and durations are different. In the study by Liou, it was expected that an increase in the number of subcutaneous injections may induce more inflammation in the animals. G-CSF has been demonstrated to have a therapeutic window (0–48 h) in this study, and it will crucially affect the long-term pain behaviors of animals with nerve injury. Thus, the G-CSF injection time-point at 72 h after CCI showed no significant effect on thermal hyperalgesia, but continued to produce inflammatory effects after that period (2) Cancer pain is a more complex and chronic process; therefore, peripheral tissue may become adapted to the stimuli and produce less CRF and fewer chemokines (ex. CXC family). Thus, the analgesic effects of G-CSF may be less apparent without sufficient CRF and CXC chemokines.

Several peripheral endogenous antinociceptive mechanisms are involved in counteracting inflammatory hyperalgesia [47]–[49]. Under inflammatory conditions, leukocytes secrete opioid peptides that bind to opioid receptors on peripheral sensory neurons and mediate antinociception [11], [13], [14], [16]. G-CSF is potentially important for the development of the immune and nervous systems, but its effects on neuropathic pain have not been fully elucidated. We suggest that high doses of G-CSF via different routes might be able to deliver its neuroprotective effects *in vivo*. Moreover, G-CSF treatment can increase the number of leukocytes [50] and enhance T-cell cytokine secretion [51], [52]. G-CSF also exerts some neuroprotective actions through the inhibition of apoptosis and inflammation as well as through the stimulation of neurogenesis [53], which may also contribute toward alleviating neuropathic pain. Alternatively, G-CSF modulates the micro-environment in inflammatory sites, including cytokine expression profiles, resulting in enhanced cell survival, proliferation, and differentiation into cells of the neural lineage of bone marrow-derived stem cells that migrate into the lesion site [54].

In the present study, G-CSF treatment increased PMN cell proliferation in the peripheral blood and injured nerve within 3–48 h and 12–48 h, respectively, of CCI rats, which was correlated with the analgesic effect on the study animals. In contrast, G-CSF significantly reduced the expression of IL-6 mRNA and TNF-α protein in the DRG at 48 and 72 h and thereafter, and attenuated microglial activation in the spinal dorsal horn 72 h after CCI. The time sequence of the effect of G-CSF on CCI rats may suggest that G-CSF recruits more opioid-containing PMN cells to the injured nerve with local opioid release and reacts with opioid receptors, thereby attenuating peripheral nociceptor signal transmission, which, in turn, decreases DRG IL-6 mRNA and TNF-α protein expression; this is followed by reduced microglial activation in the spinal dorsal horn (Fig. 8). Thus, opioid-containing PMN cells recruited by G-CSF play an essential role in analgesic effects in CCI rats. Alternatively, we cannot exclude the possibility that G-CSF may hinder the progression of inflammation at multiple sites [55]; for example, by directly mediating a decrease in proinflammatory cytokines in the injured nerve and DRG, and microglial activation in the spinal dorsal horn.

The treatment of severe pain with opioids is limited by their undesirable central side effects. This study shows that exogenously applied recombinant human G-CSF is a promising new therapeutic avenue for treating neuropathic pain by providing opioid analgesics that act outside the central nervous system, targeting opioid peptide-containing immune cells to injured peripheral nerves, and enhancing opioid production at the sites of injury while not being hampered by side effects such as endogenesis or opioid-induced addiction.
